# Rheumatology in Africa—challenges and opportunities

**DOI:** 10.1186/s13075-017-1259-3

**Published:** 2017-03-07

**Authors:** Girish M. Mody

**Affiliations:** Department of Rheumatology, School of Clinical Medicine, University of KwaZulu-Natal and Inkosi Albert Luthuli Central Hospital, 719 Umbilo Road, Durban, 4001 South Africa

**Keywords:** Rheumatology, Africa, Blacks, Arthritis, Health equity, Rheumatoid arthritis, Genetics

## Abstract

Africa faces many health challenges despite sustained growth and development over the past decade. Contributory factors are the lack of financial resources, an inadequate health professional workforce, a high burden of communicable diseases, and an increasing burden of non-communicable diseases. Rheumatology services are limited or non-existent in many parts of sub-Saharan Africa. Over the past decade, partnerships with international academic institutions have resulted in some progress in the training of rheumatologists and health professionals and development of rheumatology services in countries such as Kenya, Nigeria, and Zambia. Basic diagnostic tests, biological agents, and arthroplasty are either unavailable or not affordable by the majority of the population. Urbanization has resulted in a change in the epidemiology of rheumatic diseases with an increase in the prevalence of gout, rheumatoid arthritis, systemic lupus erythematosus, and scleroderma over the past four decades. Future growth of rheumatology services will depend on identifying committed individuals in underserved countries for training and supporting them to educate medical students, physicians, and health professionals in their home countries. There is a need to develop models of care using all categories of health workers and identify prevention strategies and cost-effective management programs for low resource settings. Africa affords an opportunity for collaborative research, including genetic and epigenetic studies, to improve our understanding of many of the rheumatic diseases.

## Background

Many global initiatives have been undertaken to address disparities in health care, especially in developing countries. In rheumatology, considerable growth has occurred in the Asia Pacific region and South America but progress has been slower in Africa, especially sub-Saharan Africa (SSA). This commentary reviews some challenges, records some of the current initiatives and progress, and identifies opportunities for further development of rheumatology in Africa.

Africa is the second most populous region in the world with an estimated 1.2 billion people in 2016. Over the past decade, SSA has shown sustained economic growth and development. Despite this progress, the challenges facing Africa are many and include limited financial resources for adequate nutrition, access to water, sanitation, housing, and education. The available health care resources have many competing priorities, including the high burden of communicable diseases and the rising prevalence of non-communicable diseases. There is a shortage of medical personnel with only 2.7 physicians per 10,000 population in Africa compared to 5.9 in South East Asia, 12.7 in the eastern Mediterranean, 15.5 in the western Pacific, 21.5 in the Americas, and 32.1 in the European region [1]. Many countries in SSA already have models of care using nurses and community health workers. A survey of 47 countries in SSA showed that 25 were already using non-physician clinicians [2]. Many academic institutions in the UK, Europe, and North America support education and training programs in Africa.

The 2010 Global Burden of Disease survey showed that rheumatic and musculoskeletal diseases have the fourth highest global impact on disability-adjusted life years and are the second leading cause of disability as measured by years lived with disability [3, 4]. Epidemiological studies have shown that although the prevalence of musculoskeletal disorders in the developing world is similar to that in the developed world, the burden is higher [5]. The increased burden is due to delayed diagnosis arising from poor education, sociocultural beliefs, poverty, and limited access to care. Although epidemiological data in Africa are limited, large studies on rheumatoid arthritis and musculoskeletal pain, including low back pain, have recently been reported [6–8]. The doubling of the population of people aged over 60 years in SSA from 23 million in 1990 to 46 million in 2015 will further increase the burden [9].

North African countries, such as Algeria, Tunisia, Morocco, and Egypt, and South Africa have a relatively larger number of rheumatologists, although South Africa has only 85 adult and pediatric rheumatologists for nearly 56 million people. The situation is worse in some countries in SSA, which have a few or even no rheumatologists or rheumatology services. In many countries, diagnostic tests, biological agents, arthroplasty, and renal replacement therapy are scarce, and usually unaffordable.

The 2013 African League of Associations for Rheumatology congress in South Africa attracted delegates from 20 African countries. A few dedicated and committed colleagues made substantial gains in the training of rheumatologists in Nigeria and Kenya with the support of international partners. Rheumatologists from North America, UK, Europe, and Africa secured grants from the International League of Associations for Rheumatology to develop rheumatology services in Zambia and Kenya [10]. Rheumatologists from Canada and the USA are currently involved in education and training in Ethiopia, where there are no rheumatologists. The UWEZO project, a collaboration between Kenyan, UK, and Swedish rheumatologists, trained a team of physicians and health workers who conducted an educational program at 11 sites across Kenya and provided basic skills to over 500 health providers [11]. Health professionals, including nurses, play an important role in the care of rheumatology patients even in developed nations. The European League Against Rheumatism has provided recommendations for the role of nurses in patients with inflammatory arthritis [12].

The future growth of rheumatology services in Africa will depend on identifying interested persons in underserved countries who have the support of their academic institutions and health ministries. Collaboration with international academic institutions will help to train more rheumatologists. These trained personnel will require support to develop rheumatology services and improve the knowledge and skills of medical students, physicians, and other health workers in their countries. There is also a need to develop strategies for prevention, diagnostics, and cost-effective intervention relevant to low resource settings.

Africa has recently shown the greatest rate of urbanization compared to the rest of the world. An effect of urbanization has been an increase in “western” diseases such as hypertension, obesity, diabetes, heart disease, and asthma [13]. Among the rheumatic diseases, there is a dramatic increase in gout, and the frequency of rheumatoid arthritis, systemic lupus erythematosus, and scleroderma has increased over the past four decades. Many countries in Africa have reported larger series of patients with rheumatoid arthritis. Although some studies report a lower prevalence of rheumatoid factor and systemic manifestations, many studies have confirmed a genetic association with HLA class II antigens. A recent Cameroon study, using 28 Caucasian susceptibility single nucleotide polymorphisms, showed different genetic susceptibility in African blacks [14]. Osteoarthritis, especially involving the knee, is the commonest cause of arthritis in Africa. Ankylosing spondylitis and psoriatic arthritis are uncommon in African blacks, and while Takayasu’s arteritis occurs in African blacks, conditions such as giant cell arteritis, polymyalgia rheumatica, and polyarteritis nodosa are extremely rare. Africa provides a unique opportunity to study the expression and outcome of rheumatic diseases in the nearly 10.3 million people with HIV who are on anti-retroviral therapy [15].

## Conclusion

Although rheumatology in Africa has made some progress, there is still a great need. If our rheumatology colleagues are able to join the outreach programs of their institutions, they can help accelerate the growth of rheumatology in Africa. Collaboration in education and research will provide an enriching experience and improve outcomes in underserved communities. The findings of fossils in Africa have contributed to the theories on the origin of man. Is it possible that genetic and epigenetic studies in Africa will identify risk or protective factors to improve our understanding of the pathogenesis of the rheumatic diseases?

## Abbreviations

SSA: Sub-Saharan Africa.

## References

[1] World Health Organization. World Health Statistics 2015. http://apps.who.int/iris/bitstream/10665/170250/1/9789240694439_eng.pdf?ua=1&ua=1. Accessed 26 Jan 2017.

[2] Mullan F, Frehywot S (2007). Non-physician clinicians in 47 sub-Saharan African countries. Lancet..

[3] Murray CJ, Vos T, Lozano R, Naghavi M, Flaxman AD, Michaud C (2012). Disability-adjusted life years (DALYs) for 291 diseases and injuries in 21 regions, 1990-2010: a systematic analysis for the Global Burden of Disease Study 2010. Lancet..

[4] Vos T, Flaxman AD, Naghavi M, Lozano R, Michaud C, Ezzati M (2012). Years lived with disability (YLDs) for 1160 sequelae of 289 diseases and injuries 1990-2010: a systematic analysis for the Global Burden of Disease Study 2010. Lancet..

[5] Chopra A, Abdel-Nasser A (2008). Epidemiology of rheumatic musculoskeletal disorders in the developing world. Best Pract Res Clin Rheumatol..

[6] Usenbo A, Kramer V, Young T, Musekiwa A (2015). Prevalence of arthritis in Africa: a systematic review and meta-analysis. PLoS One..

[7] Slimani S, Ladjouze-Rezig A (2014). Prevalence of rheumatoid arthritis in an urban population of Algeria: a prospective study. Rheumatology (Oxford).

[8] Louw QA, Morris LD, Grimmer-Somers K (2007). The prevalence of low back pain in Africa: a systematic review. BMC Musculoskelet Disord..

[9] United Nations, Department of Economic and Social Affairs, Population Division (2015). World Population Ageing.

[10] Genga EK, Moots RJ, Oyoo OG, OOtieno F (2016). Building a rheumatology team for East Africa: a call for action! Rheumatology (Oxford).

[11] Erwin J, Woolf A, Oyoo O, Cederlund I, Mwaniki L, Etau P (2016). The UWEZO project-musculoskeletal health training in Kenya. Clin Rheumatol..

[12] van Eijk-Hustings Y, van Tubergen A, Bostrom C, Braychenko E, Buss B, Felix J (2012). EULAR recommendations for the role of the nurse in the management of chronic inflammatory arthritis. Ann Rheum Dis..

[13] Godfrey R, Julien M (2005). Urbanisation and health. Clin Med (Lond).

[14] Viatte S, Flynn E, Lunt M, Barnes J, Singwe-Ngandeu M, Bas S (2012). Investigation of Caucasian rheumatoid arthritis susceptibility loci in African patients with the same disease. Arthritis Res Ther..

[15] Joint United Nations Programme on HIV/AIDS (UNAIDS) Global AIDS update 2016. Geneva, Switzerland. 2016. http://www.unaids.org/sites/default/files/media_asset/global-AIDS-update-2016_en.pdf. Accessed 26 Jan 2017.

